# Impaired Ca^2+^ release contributes to muscle weakness in a rat model of critical illness myopathy

**DOI:** 10.1186/s13054-016-1417-z

**Published:** 2016-08-10

**Authors:** Monica Llano-Diez, Arthur J. Cheng, William Jonsson, Niklas Ivarsson, Håkan Westerblad, Vic Sun, Nicola Cacciani, Lars Larsson, Joseph Bruton

**Affiliations:** Department of Physiology & Pharmacology, Karolinska Institutet, von Eulers väg, 8, 2 floor, Stockholm, 171 77 Sweden

**Keywords:** Critical illness myopathy, Intensive care unit, Skeletal muscle weakness, Ca^2+^ handling, Excitation-contraction coupling

## Abstract

**Background:**

Critical illness myopathy is an acquired skeletal muscle disorder with severe myosin loss and muscle weakness frequently seen in intensive care unit (ICU) patients. It is unknown if impaired excitation-contraction coupling contributes to the muscle weakness.

**Methods:**

We used a unique ICU model where rats were deeply sedated, post-synaptically pharmacologically paralyzed, mechanically ventilated and closely monitored for up to ten days. Single intact fibers from the flexor digitorum brevis muscle were isolated and used to measure force and free myoplasmic [Ca^2+^] ([Ca^2+^]_i_) during tetanic contractions.

**Results:**

Fibers from ICU rats had 80 % lower tetanic [Ca^2+^]_i_ and produced only 15 % of the force seen in fibers from sham-operated (SHAM) rats. In the presence of 5 mM caffeine, tetanic [Ca^2+^]_i_ was similar in fibers from ICU and SHAM rats but force was 50 % lower in fibers from ICU rats than SHAM rats. Confocal imaging showed disrupted tetanic [Ca^2+^]_i_ transients in fibers from ICU rats compared to SHAM rats. Western blots showed similar levels of Na^+^ channel and dihydropyridine receptor (DHPR) protein expression, whereas ryanodine receptor (RyR) and sarco-endoplasmic reticulum Ca^2+^ ATPase 1 (SERCA1) expression was markedly lower in muscle of ICU rats than in SHAM rats. Immunohistochemical analysis showed that distribution of Na^+^ channel and DHPR protein on the sarcolemma was disrupted in fibers from ICU rats compared with SHAM rats.

**Conclusions:**

These results suggest that impaired SR Ca^2+^ release contributes to the muscle weakness seen in patients in ICU.

**Electronic supplementary material:**

The online version of this article (doi:10.1186/s13054-016-1417-z) contains supplementary material, which is available to authorized users.

## Background

Critical illness myopathy (CIM) is a neuromuscular disorder frequently observed in intensive care unit (ICU) patients exposed to long-term mechanical ventilation and immobilization. It is characterized by severe muscle weakness in limb and respiratory muscles, muscle wasting and a preferential loss of myosin and myosin-associated proteins [1–5]. Treatment of patients who develop CIM is expensive and CIM is associated with higher mortality rates [6].

The mechanisms that underlie the altered muscle function in ICU patients are incompletely understood [5, 7]. Recently, a rat model was introduced that mimics the ICU conditions of long-term sedation, mechanical ventilation and muscle unloading but avoids confounding factors related to age, gender, pre-existing disease or drug treatment. This ICU model develops a phenotype that closely resembles the key features of CIM in humans with loss of myosin, dysregulation of myofibrillar protein turnover, muscle atrophy and loss of specific force [8, 9].

The myosin loss and muscle atrophy in CIM will lead to muscle weakness. However, it is currently unknown whether intrinsic defects in excitation-contraction coupling (ECC) such as changes in action potentials or Ca^2+^ release and re-uptake by the sarcoplasmic reticulum (SR) also contribute to the muscle weakness [5, 10]. It is reported that in critically ill patients and in animal models the skeletal muscles have reduced muscle membrane excitability due to either depolarization and/or inactivated Na^+^ channels [11–14]. The dihydropyridine receptor (DHPR) communicates depolarization of the sarcolemma to the ryanodine receptors (RyR) in the sarcoplasmic reticulum (SR) membrane causing them to open and release Ca^2+^ into the myoplasm. However, there are few data on changes in DHPR or RyR protein or function aside from one study, which showed increased DHPR and RyR1 protein expression in skeletal muscle in an ICU rat model [15]. To date, there are no data on intracellular Ca^2+^ transients evoked by action potentials in the muscles of patients on ICU. In the present study, we hypothesized that in skeletal muscle from the ICU model rats, ECC is impaired and this contributes to the muscle weakness. Our results show clearly that in muscle fibers from rats subjected to 8–10 days of ICU simulation, action potential-induced SR Ca^2+^ release is significantly reduced.

## Methods

### Ethics statement

The ethical committee at Uppsala University approved all aspects of this study and it complied with the recommendations in the *Guide for the Care and Use of Laboratory Animals* [16].

### Animals and tissue collection

Eight sham-operated controls (hereafter called SHAM) and seven experimental female Sprague–Dawley rats (weight: 235–299 g, Taconic, Denmark) were included in this study. The experimental (hereafter called ICU) rats were anesthetized with isoflurane, treated with the neuromuscular blocker α-cobratoxin and mechanically ventilated for up to 10 days as described in detail previously [17]. Briefly, the following preparatory surgery was carried out under sterile conditions. Precordial silver wire electrocardiogram (ECG) electrodes were implanted subcutaneously. Arterial blood pressure was monitored with an aortic catheter (28-gauge Teflon) inserted into the left carotid artery. A 0.9-mm Renathane catheter was threaded into the left jugular vein to administer parental solutions. Three subcutaneous electroencephalogram (EEG) needle electrodes were placed into the skull above the right and left temporal lobes, and a third reference electrode was placed in the neck region to record brain activity. Temperature was measured by a vaginal thermistor and servo-regulated at 37 °C. A silicone cannula was inserted in the urethra to continuously record urine output. SHAM rats underwent the same interventions as the ICU rats, except that they were not pharmacologically paralyzed with α-cobratoxin and breathed spontaneously. SHAM rats were killed within 2 hours after the anesthesia had started.

During surgery or other potentially irritating interventions, the isoflurane level was kept at >1.5 %. After the initial surgery, the isoflurane concentration was gradually reduced over the next 1–2 days and kept at <0.5 % for the rest of the experiment. Rats were ventilated through a coaxial tracheal cannula at 72 breaths/minute with an inspiratory-expiratory ratio of 1:2 and a minute volume of 180–200 ml and gas concentrations of 50 % O_2_, 47 % N_2_, and 3 % CO_2_. Expiratory CO_2_ was monitored continuously. Intermittent respiratory hyperinflations (six per hour at 15 cm H_2_O) were delivered throughout the experiment. Neuromuscular blockade was induced (100 μg α-cobratoxin injected intravenously) and maintained by continuous infusion (250 μg/day intravenously). Mechanical ventilation was initiated immediately after the neuromuscular blocker induction. Experiments were terminated after eight or ten days. In no case did the anesthetized rats show any sign of infection, septicemia or systemic inflammation.

Immediately after euthanasia by removal of the heart, the flexor digitorum brevis (FDB) and tibialis anterior (TA) muscles were dissected from both legs. The TA muscles were quickly frozen in liquid propane pre-cooled in liquid nitrogen and stored at −160 °C for further analyses. The FDB muscles were placed in Tyrode solution supplemented with 0.2 % fetal bovine serum and transported to the laboratory for single fiber isolation.

### Specific force and [Ca^2+^]_i_ measurements

The FDB muscles were split into individual digits and the central three digits were used in these experiments. Fibers were stimulated with a handheld homemade point stimulation electrode that delivered 1 ms, 6–10 V pulses. The electrode was used to stimulate fibers at various points along their length. The overwhelming majority of fibers from SHAM rats contracted when stimulated at any point along their length. In contrast, less than half of the fibers from ICU rats contracted. Using iris scissors and jeweler’s forceps we dissected only those FDB fibers that contracted when electrically stimulated. Aluminum or platinum T-clips were attached to the tendons of the isolated fiber. The fiber was then placed horizontally in a superfusion chamber between a force transducer (AE801, Kronex Technologies, CA, USA) and an adjustable holder. The fiber was stimulated with supramaximal electrical pulses (0.5–1 ms in duration) delivered via platinum electrodes placed parallel to the long axis of the fiber. The length of the fiber was adjusted until tetanic force was maximal, and the fiber diameter at this length was measured to calculate the cross-sectional area.

The mean diameter of dissected fibers was 35 ± 1 and 30 ± 1 for fibers from SHAM and ICU rats, respectively. Tetanic force was normalized to cross-sectional area and expressed as specific force (kN/m^2^). Fibers were superfused with Tyrode solution containing (in mM) 121 NaCl, 5.0 KCl, 1.8 CaCl_2_, 0.5 MgCl_2_, 0.4 NaH_2_PO_4_, 24.0 NaHCO_3_, 0.1 EDTA, and 5.5 glucose at room temperature (24–26 °C). Fetal bovine serum (0.2 %) was added to the solution. The solution was bubbled with 95 % O_2_ - 5 % CO_2_, giving a pH of 7.4. When used, 5 mM caffeine was dissolved in Tyrode and super-fused over the fiber for one minute before electrical stimulation.

To measure resting and tetanic [Ca^2+^]_i_, fibers were pressure injected with the fluorescent indicator indo-1 (Molecular Probes/Invitrogen, Carlsbad, CA, USA). Indo-1 was excited at 360 ± 5 nm and the light emitted at 405 ± 5 and 495 ± 5 nm was measured with a pair of photomultiplier tubes (Photon Technology International, Wedel, Germany). Indo-1 fluorescence signals were converted into [Ca^2+^]_i_ as described previously [18]. Mean resting [Ca^2+^]_i_ was measuring during the 200 ms preceding a tetanus. Tetanic [Ca^2+^]_i_ was measured as the mean of the final 250 ms of the 350 ms tetanus, and tetanic force was measured as the peak force during the tetanus. Fluorescence and force signals were sampled online and stored on a computer for subsequent data analyses.

In some experiments, enzymatically isolated FDB fibers were loaded with the fluorescent [Ca^2+^]_i_ indicator fluo-3 (4 μM, 20 minutes at room temperature) and spatial and temporal aspects of [Ca^2+^]_i_ transients were examined during 70 Hz electrical stimulation. A BioRad MRC 1024 confocal unit equipped with a Calypso laser (Cobolt, Solna, Sweden) attached to a Nikon Diaphot 200 inverted microscope with a Nikon Plan Apo × 20 objective lens was used to obtain line scan images (6 ms per line) of changes in the fluo-3 signal. The fiber was placed to ensure that the line scan was performed across the diameter of the fiber. Fluo-3 was excited at 491 nm, and the emitted light was collected through a 515 long-pass filter. Images were analyzed using ImageJ software (National Institutes of Health, Bethesda, MD, USA; https://imagej.nih.gov/ij/). Background fluorescence was subtracted and the fluorescence during electrically induced contractions is expressed relative to that measured before electrical stimulation (F/F_0_).

### Repeated stimulation protocol

Fatigue was induced by stimulating fibers with 150 Hz, 500 ms tetani repeated at 1 s intervals until force decreased to 40 % of the initial force. Fibers from ICU rats contracted very weakly and to get a sizeable force at the start of fatigue runs, these fibers were exposed to 5 mM caffeine for one minute and then fatigued in the continued presence of caffeine [19].

### Western blot

Isolated muscles were homogenized with a ground glass homogenizer in ice-cold homogenisation buffer at pH 7.4 (20 μl per mg wet weight) consisting of (mM): Hepes, 20; NaCl, 150; EDTA, 5; KF, 25; Na_3_VO_4_, 1; and 20 % glycerol, 0.5 % Triton X-100, and protease inhibitor cocktail (Roche, Basel, Switzerland), one tablet/50 ml. The homogenate was centrifuged at 700 g for 10 minutes at 4 °C. Protein content of the supernatant was determined using the Bradford assay (#500-0006, Bio-Rad, Hercules, CA, USA). Samples were diluted 1:1 in Laemmli buffer (Bio-Rad) with 5 % 2-mercaptoethanol and heated to 95 °C for 5 minutes.

Ten μg protein was run on a 4–12 % precast Bis–Tris gel (NP0336PK2, NuPAGE, Invitrogen Carlsbad, CA, USA) and transferred onto polyvinylidine fluoride membranes (Immobilon FL, Millipore, Billerica, MA, USA). Membranes were then blocked with Li-Cor Blocking buffer (LI-COR Biosciences, Lincoln, NE, USA) followed by incubation overnight at 4 °C with the following antibodies diluted in blocking buffer; mouse anti-dihydropyridine receptor (DHPR; Abcam, Cambridge, UK, ab2864, 1:1000), mouse anti-ryanodine receptor 1(RyR1; Abcam, ab2868, 1:1000), anti-Na^+^ channel (Millipore, Catalog number 06-811, 1:1000), anti-sarco-endoplasmic reticulum Ca^2+^ ATPase 1 (SERCA1; Abcam, ab109899, 1:1000). Membranes were then washed and incubated with secondary antibody IRDye 680-conjugated donkey anti-mouse IgG and IRDye 800-conjugated donkey anti-rabbit IgG (926–68072, 926–32213, LI-COR). Immunoreactive bands were visualized using infrared fluorescence (IR-Odyssey scanner, LI-COR Biosciences). Band density was measured using Image Studio v 2.0.38 (LI-COR Biosciences) and normalized to subsequent Coomassie protein staining (Additional file 1: Figure S1) of the same membranes (#161-0436, Bio-Rad).

### Immunohistochemical analysis

Cross-sections or longitudinal sections, 10 μm thick, were cut from the tibialis anterior (TA) muscle in a cryostat at −23 °C and placed on glass slides. Longitudinal sections were used to visualize the distribution of Na^+^ channels and DHPR in the sarcolemma while transverse sections were preferred to identify the distribution of the RyR and SERCA1 proteins embedded in the sarcoplasmic reticulum membrane. The tissue sections were air dried at room temperature and stored at −80 °C. The sections were fixed for 20 minutes in cold 2 % formaldehyde diluted in phosphate-buffered saline system (PBS) (Sigma-Aldrich), and then washed with 0.1 % saponin (Sigma) at pH 7.4 (referred to as PBS-Sap) for 10 minutes. The blocking step was performed with 5 % normal goat serum (Sigma-Aldrich) for 1 hour at room temperature. After washing with PBS-Sap, sections were stained with anti-ryanodine receptor 1 (RyR1; Abcam, ab2868, 1:600), anti-sarco-endoplasmic reticulum Ca^2+^ ATPase 1 (SERCA1; Abcam, ab109899, 1:200), anti-Na^+^ channel (Millipore, Catalog number 06-811, 10 μg/ml), and anti-DHPR (Abcam, ab2864, 1:600) diluted in 1 % goat serum + PBS-Sap and incubated overnight at 4 °C in a humid chamber. After washing with PBS-Sap, secondary antibodies (Alexa 488 goat anti-rabbit IgG, or Alexa 488 goat anti-mouse IgG, 1:1000, Invitrogen, Oregon, USA) diluted in 1 % goat serum + PBS-Sap were applied to the sections for 30 minutes in the dark. A negative control for each run was used (Additional file 2: Figure S2). Slides were mounted using the anti-fade mounting medium for fluorescence Vectashield (Vector, Burlingame, CA). Slides were examined using a Bio-Rad MRC 1024 confocal unit with a Calypso dual laser (Cobolt, Solna, Sweden) attached to a Nikon Diaphot 200 inverted microscope and a Nikon Plan Apo × 40 oil-immersion objective, N.A. 1.3 (Nikon, Tokyo, Japan). The stained sections were coded and evaluated in a descriptive manner by independent observers (MLD, JDB, VS and AJC). Images were prepared using ImageJ software (National Institutes of Health, Bethesda, MD; https://imagej.nih.gov/ij/).

### Statistical analysis

Data are presented as mean ± SEM. Statistical analyses were performed using the SigmaPlot software (Systat Software, Inc., CA, USA). Unpaired *t* tests were performed to establish significant differences between groups and a *p* value <0.05 was considered to be statistically significant.

## Results

### Tetanic [Ca^2+^]_i_ and specific force in FDB fibers are reduced after 8–10 days of the ICU intervention

**Fig. 1:**
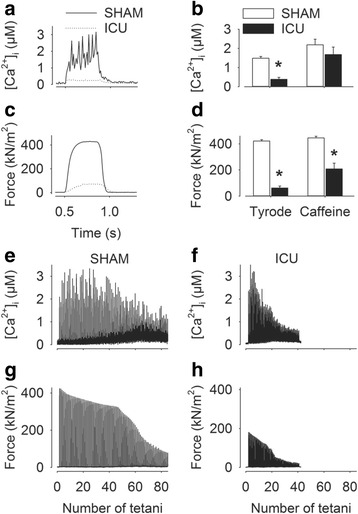
Tetanic free myoplasmic [Ca^2+^] (*[Ca* ^*2+*^ *]*_*i*_) and specific force are markedly lower in fibers from ICU rats. Typical tetanic [Ca^2+^]_i_ (**a**) and specific force transients (**c**) in fibers from sham-operated (SHAM) (*solid traces*) and ICU rats (*dotted traces*). Mean ± SEM values for tetanic [Ca^2+^]_i_ (**b**) and specific force (**d**) in fibers from SHAM rats (*open bars*, n = 6) and ICU rats (*solid bars*, n = 9) in Tyrode only, or in the presence of 5 mM caffeine. *p < 0.05 ICU vs SHAM rats. **e**-**f** Representative records of [Ca^2+^]_i_ (**e**, **f**) and specific force (**g**, **h**) during fatigue induced by repeated tetani in fiber from a SHAM rat (**e**, **g**) and an ICU rat (**f**, **h**)

Resting [Ca^2+^]_i_ was not significantly different in fibers from SHAM and ICU rats (73 ± 4 nM (n = 6) and 61 ± 7 nM (n = 9), respectively). Figure 1a shows that tetanic [Ca^2+^]_i_ was considerably lower in a fiber from an ICU rat (dotted trace) than in a fiber from a SHAM rat (solid trace). The resultant specific tetanic force was also smaller in the fiber from the ICU rat than in that from the SHAM rat (Fig. 1c). Mean data show clearly that both tetanic [Ca^2+^]_i_ (Fig. 1b) and specific force (Fig. 1d) were markedly smaller (*p* < 0.05) in fibers from ICU compared to SHAM rats.

To determine whether the lower tetanic [Ca^2+^]_i_ and specific force recorded fibers from ICU rats were due to less Ca^2+^ being stored in the SR, the fibers were electrically stimulated in the presence of 5 mM caffeine, which potentiates SR Ca^2+^ release and releases all stored free Ca^2+^ [19]. The presence of caffeine significantly increased tetanic [Ca^2+^]_i_ (*p* < 0.05) in fibers from both ICU and SHAM rats and there was no longer any statistically significant difference in tetanic [Ca^2+^]_i_ between fibers from ICU and SHAM rats (Fig. 1b), indicating that SR Ca^2+^ stores were not markedly reduced in fibers from ICU compared to SHAM rats. However, despite the marked increased in tetanic [Ca^2+^]_i_ induced by the presence of caffeine, mean specific tetanic force was still approximately 50 % lower ((Fig. 1d, *p* < 0.05) in fibers from ICU than in SHAM rats, reflecting the fact that maximum force-generating capacity was lower in the fibers from ICU rats than in the SHAM rats.

Some fibers were fatigued by repeated tetanic stimulation and fibers from ICU rats underwent this stimulation in the presence of caffeine in order to have a sizeable starting force. Figure 1e-h shows representative records of [Ca^2+^]_i_ (Fig. 1e, f) and specific force (Fig. 1g, h) during repetitive stimulation of fibers from SHAM and ICU rats. The fiber from ICU rats clearly responded to repeated tetanic stimulation, but the amplitude of [Ca^2+^]_i_ and force transients decreased more rapidly than those in the fiber from SHAM rats. The changes in resting [Ca^2+^]_i_ during the period of repetitive stimulation was similar in fibers from SHAM and ICU rats indicating that SR Ca^2+^ pumping increased in both the ICU and the SHAM rats. Overall, these results show that fibers from ICU rats increase ATP production to cope with the increased energy demands of repeated tetanic [Ca^2+^]_i_ and force transients.

**Fig. 2:**
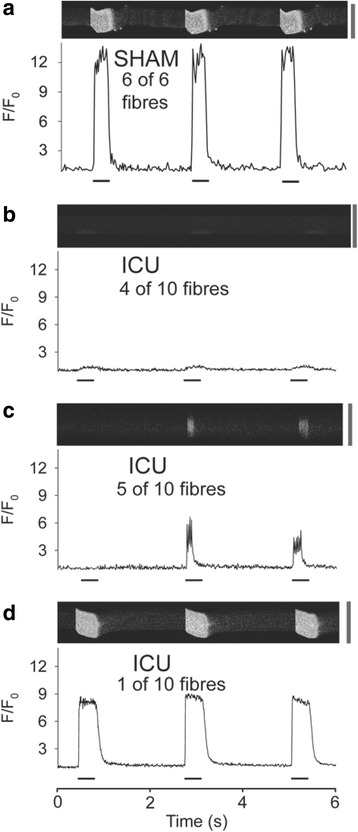
Ca^2+^ release from the sarcoplasmic reticulum is reduced and non-uniform in muscle fibers from ICU rats. Panels **a**-**d** show representative line scans of fluo-3 fluorescence from flexor digitorum brevis (FDB) fibers during three 70-Hz tetani at 2-s intervals. **a** Typical homogeneous free myoplasmic [Ca^2+^] ([Ca^2+^]_i_) transient during each of the three tetani seen in sham-operated (SHAM) rat fibers. **b**-**d** The [Ca^2+^]_i_ transients observed in ICU rat fibers. **b** Ca^2+^ release is seen only at one edge of the fiber. **c** The [Ca^2+^]_i_ transients do not occur in response to each tetanic stimulation and do not last throughout the whole period of electrical stimulation. **d** The [Ca^2+^]_i_ transients occurred during each of the three tetani but were reduced compared to those in SHAM rat fibers. Fluorescence intensity (*F*) is expressed relative to the value at rest (*F*_*0*_). Periods of electrical stimulation are indicated by the *black bars* underneath each trace. *Calibration bar* to the *right* of each line scan is 50 μm

Confocal line scans of the FDB fibers were used to determine if SR Ca^2+^ release was homogenous in fibers during three repeated tetani. Figure 2a shows the typical response in a fiber from a SHAM rat where [Ca^2+^]_i_ increased uniformly throughout the fiber during each tetanic stimulation. Similar results were observed in a further five fibers from two other SHAM rats. In contrast to SHAM fibers, the majority of fibers from four ICU rats had severe defects in SR Ca^2+^ release with [Ca^2+^]_i_ increasing only close to the surface of the fiber (four of ten fibers, Fig. 2b) or with non-uniform and variable [Ca^2+^]_i_ increases upon repeated tetanic stimulation (five of ten fibers, Fig. 2c). In a minority of fibers from ICU rats (one of ten fibers, Fig. 2d), each of the three repeated tetani evoked homogenous SR Ca^2+^ release, albeit of lower amplitude than that observed in SHAM rat fibers.

### Examination of ECC proteins in SHAM and ICU rat muscle

**Fig. 3:**
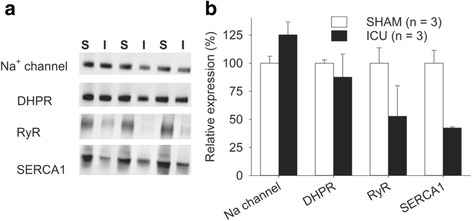
Protein expression of Na^+^ channel and dihydropyridine receptor (*DHPR*) is unchanged but protein expression of ryanodine receptor (*RyR*) and sarco-endoplasmic reticulum Ca^2+^ ATPase 1 (*SERCA1*) is markedly lower in ICU rat muscle than in sham-operated (SHAM) rat muscle. **a** Typical protein bands from SHAM (*S*) or ICU (*I*) rats for Na^+^ channels, DHPR, RyR1and SERCA1. **b** Mean data for intensity of bands expressed relative to total intensity of each corresponding lane. Values are mean ± SEM, n = 3 in all cases

**Fig. 4:**
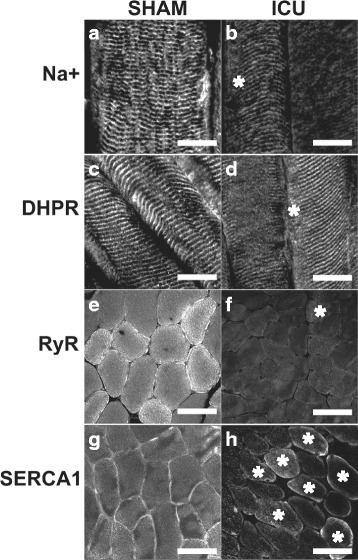
Immunofluorescent staining of sham-operated (SHAM) rat and ICU rat muscle samples. Longitudinal sections of rat tibialis anterior (TA) muscle stained with antibodies to Na^+^ channels (**a**, **b**) or dihydropyridine receptor (*DHPR*) (**c**, **d**) in SHAM rat and ICU rat muscles. The discrete and regular white punctate staining in SHAM muscle fibers is less obvious in ICU rat muscle fibers. **e**-**f** Cross-sections of SHAM rat (**e**, **g**) and ICU rat (**f**, **h**) muscles stained for ryanodine receptor (*RyR*) (**e**, **f**) or sarco-endoplasmic reticulum Ca^2+^ ATPase 1 (*SERCA1*) (**g**, **h**) proteins. Majority of SHAM rat muscle fibers show *gray* or *bright* staining closer to the sarcolemma. In contrast, staining is less bright and less extensive in ICU rat muscle fibers. Images are representative of three TA muscles from SHAM and ICU rats, respectively. *White asterisks* (**b**, **d**, **f**, **h**) indicate fibers that were considered examples of positive staining. *Scale bar*, *bottom right* (**a**-**d**) represents 20 μm and 40 μm (**e**-**h**)

The decreased SR Ca^2+^ release observed in muscle fibers from ICU rats suggests that some step in ECC is impaired. As Na^+^ channels are responsible for transmitting the action potentials into the transverse tubule and causing a conformational change in the DHPR, which in turn open up the RyR in the sarcoplasmic reticulum, we examined the expression of these three proteins. The western blot data shown in Fig. 3 indicate that there were similar levels of protein expression of Na^+^ channels and DHPR protein in SHAM rat and ICU-rat muscle. We used immunofluorescence confocal microscopy to determine in longitudinal sections if the localization of Na^+^ channels and DHPR was similar in ICU-rat and SHAM rat muscle. In the ICU rat sections, some fibers had a Na^+^ channel distribution pattern similar to that of SHAM rat muscle (compare Fig. 4a and b). However, about 61 % of the fibers from the ICU rat had a lesser, more disorganized pattern of staining for Na^+^ channels. The distribution pattern of DHPR protein was rather similar, with 62 % of the fibers from the ICU rats (Fig. 4d) having lesser and more disorganized staining for DHPR protein than was found in SHAM rat fibers (Fig. 4c).

The western blot data in Fig. 3 show that protein expression level of the SR Ca^2+^-release channel, RyR, and the SR Ca^2+^ pump, SERCA1 were markedly reduced in ICU rat compared to SHAM rat muscle. This finding was confirmed by the immunofluorescence staining, which shows that both the extent and the number of fibers with clear positive staining (marked with white asterisks) for RyR1 or SERCA1 was markedly lower in ICU than in SHAM muscle fibers (Fig. 4 e**-**h).

## Discussion

Previous studies have clearly demonstrated that in critically ill patients and in ICU rat models there is a massive preferential loss of myosin from the muscles [5, 20]. In addition to this myosin loss, the present study shows clearly that in an ICU rat model the muscle fibers respond to electrical stimulation with small and non-uniform tetanic [Ca^2+^]_i_ transients that underlie the impaired force production.

The decreased tetanic [Ca^2+^]_i_ transients in ICU rat muscle fibers led us to hypothesize that proteins involved in one or more steps in ECC were affected. The decreased protein expression of the RyR receptor shown in our western blots (Fig. 3) is likely to contribute to the smaller tetanic [Ca^2+^]_i_ transients in ICU rat fibers. Stores of SR Ca^2+^, which provide the driving force for Ca^2+^ to leave the SR did not seem to be significantly different between ICU and SHAM rat muscle as caffeine, which augments SR Ca^2+^ release, increased tetanic [Ca^2+^]_i_ to similar levels in ICU and SHAM rat fibers. There are few existing data on changes in RyR in CIM or ICU models. Increased RyR immunostaining has been reported in a combined surgical denervation-steroid treatment rat model [15]. It should be noted that this model involves both partial denervation and sustained motor activity and differs markedly from both patients in ICU who develop CIM and the normally innervated, unloaded rat ICU model used in the present study.

The decreased tetanic [Ca^2+^]_i_ in ICU fibers measured during electrical stimulation might also be related to impaired activation of RyR. Earlier electrophysiological data indicate that there is decreased membrane excitability, reflecting reduced expression/activation of Na^+^ channels in the sarcolemma in patients, [11, 12]. Our western blot data show that protein expression of the Na^+^ channel is similar in ICU and SHAM rat muscles. On first observation, this may appear puzzling. However, the western blot data indicate only that the denatured Na^+^ channel is present in the muscle, and tell us nothing about the state of these channels or how they are distributed along the sarcolemmal or transverse tubule membrane in the intact muscle fibers.

The immunofluorescent images show clearly that the distribution of the Na^+^ channel staining is not normal in fibers from ICU rats, with only approximately 40 % of the muscle fibers having a pattern resembling that seen in SHAM rat fibers. It can be speculated that a variable and abnormal distribution of Na^+^ channels in the sarcolemma of ICU rat fibers leads to a variety of action potential transmission problems and ultimately impaired Ca^2+^ release from the SR as reflected by the reduced tetanic [Ca^2+^]_i_ transients recorded with indo-1. In addition, the confocal images showed that in a majority of ICU rat muscle fibers, there was an abnormal uneven spatial release of Ca^2+^ from the SR. In about 40 % of ICU fibers, ECC operated normally close to the sarcolemma but deeper in the fiber; there was clearly no SR Ca^2+^ release in ICU fibers. This is suggestive of subtle derangement of the DHPR or Na^+^ channel function in the transverse tubules.

Western blot data showed that ICU rat fibers had markedly decreased expression of SERCA1 (Fig. 3). However, the resulting reduction in total SR Ca^2+^ pumping capacity in ICU rat fibers did not have any major functional impact, as first, the resting [Ca^2+^]_i_ and Ca^2+^ stored in the SR were similar in ICU and SHAM rat fibers, and second, [Ca^2+^]_i_ rapidly returned to the baseline level when ICU rat fibers were subjected to repeated tetanic contractions (see Fig. 1).

A further contributor to the decreased tetanic [Ca^2+^]_i_ in ICU rat fibers that must be considered is the DHPR protein, either in terms of quantity or its ability to respond to depolarization by the conformational change in its protein structure that is required to open the RyR channel. It is well-known that the DHPR can exist in either an active state where it responds to depolarization, or in an inactivated/blocked state, which does not respond to depolarization, e.g., as previously described [21]. As pointed out by Friedrich and colleagues [5], aside from a study using a denervation-steroid treatment rat model [15], there have been no previous investigations of DHPR function in an ICU model. Our western blot data suggest that DHPR protein expression is not greatly different in ICU and SHAM rat muscle, while immunofluorescent images indicate that the pattern of DHPR staining differs in fibers from ICU rats compared to those from SHAM rats.

The present study focused only on those fibers that contracted upon electrical stimulation and did not examine those fibers that were electrically unexcitable. Under carefully controlled experimental conditions, the majority of ICU rat muscle fibers developed marked disturbances in excitation-contraction coupling that results in reduced amplitude and duration of tetanic [Ca^2+^]_i_ transients and lower force generation. The confocal images showed that there were marked differences in tetanic [Ca^2+^]_i_ transients in different fibers taken from the same rat. The precise reasons for this variability in response remain unclear to us. Differences in the degree of expression or localization of the Na^+^ channel or DHPR in individual fibers and/or their functional response to changes in the membrane potential are likely to underpin this variability in tetanic [Ca^2+^]_i_ transient responses. It is unlikely that a difference in fiber types between SHAM rat and ICU rat muscle plays any role. Rat FDB muscles are composed predominantly of type II fibers (almost 90 %) and while they develop significant atrophy after 2 weeks of hind limb unloading, they show no evidence of fiber transformation [22].

## Conclusions

In conclusion, our data demonstrate that in addition to the myosin loss, defective ECC due to impaired activation of RyR, and less action-potential-induced SR Ca^2+^ release contribute to the muscle weakness observed in ICU rats.

## Abbreviations

[Ca^2+^]_i_, free myoplasmic [Ca^2+^]; CIM, critical illness myopathy; DHPR, dihydropyridine receptor; ECC, excitation-contraction coupling; ECG, electrocardiogram; FDB, flexor digitorum brevis; ICU, intensive care unit; PBS, phosphate-buffered saline; RyR1, ryanodine receptor; SERCA1, sarco-endoplasmic reticulum Ca^2+^ ATPase 1; SHAM, sham-operated; SR, sarcoplasmic reticulum; TA, tibialis anterior

## Additional files

Additional file 1: Figure S1. Coomassie protein staining. Membranes used for western blot analysis of Na^+^ channels, DHPR (*left panel*), RyR1and SERCA1 (*right panel*). Lanes are marked as SHAM (*S*) or ICU (*I*); the value *110 kDa* refers to the protein marker size in the ladder lane (PDF 5931 kb)
Additional file 2: Figure S2. Typical examples of the negative controls obtained for Na^+^ channels, DHPR, RyR and SERCA1 immunohistochemical staining (PDF 871 kb)
