# Supplementary figures and images for: Impaired Ca2+ release contributes to muscle weakness in a rat model of critical illness myopathy
Source: Crit Care. 2016 Aug 10;20:254. doi: 10.1186/s13054-016-1417-z (PMC5050561; doi:10.1186/s13054-016-1417-z)

S I S I S I

110  
kDa

110  
kDa

S I S I S I

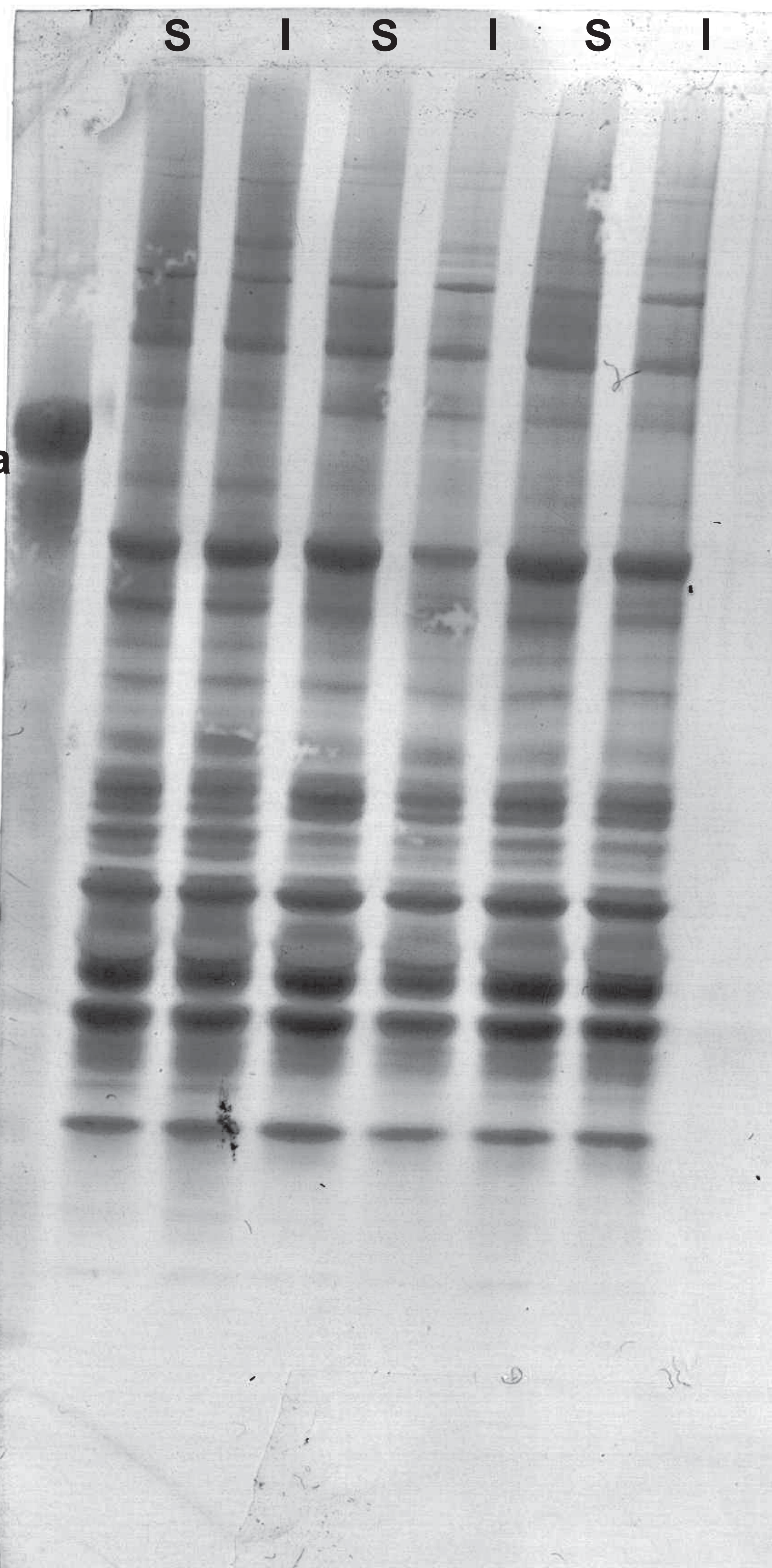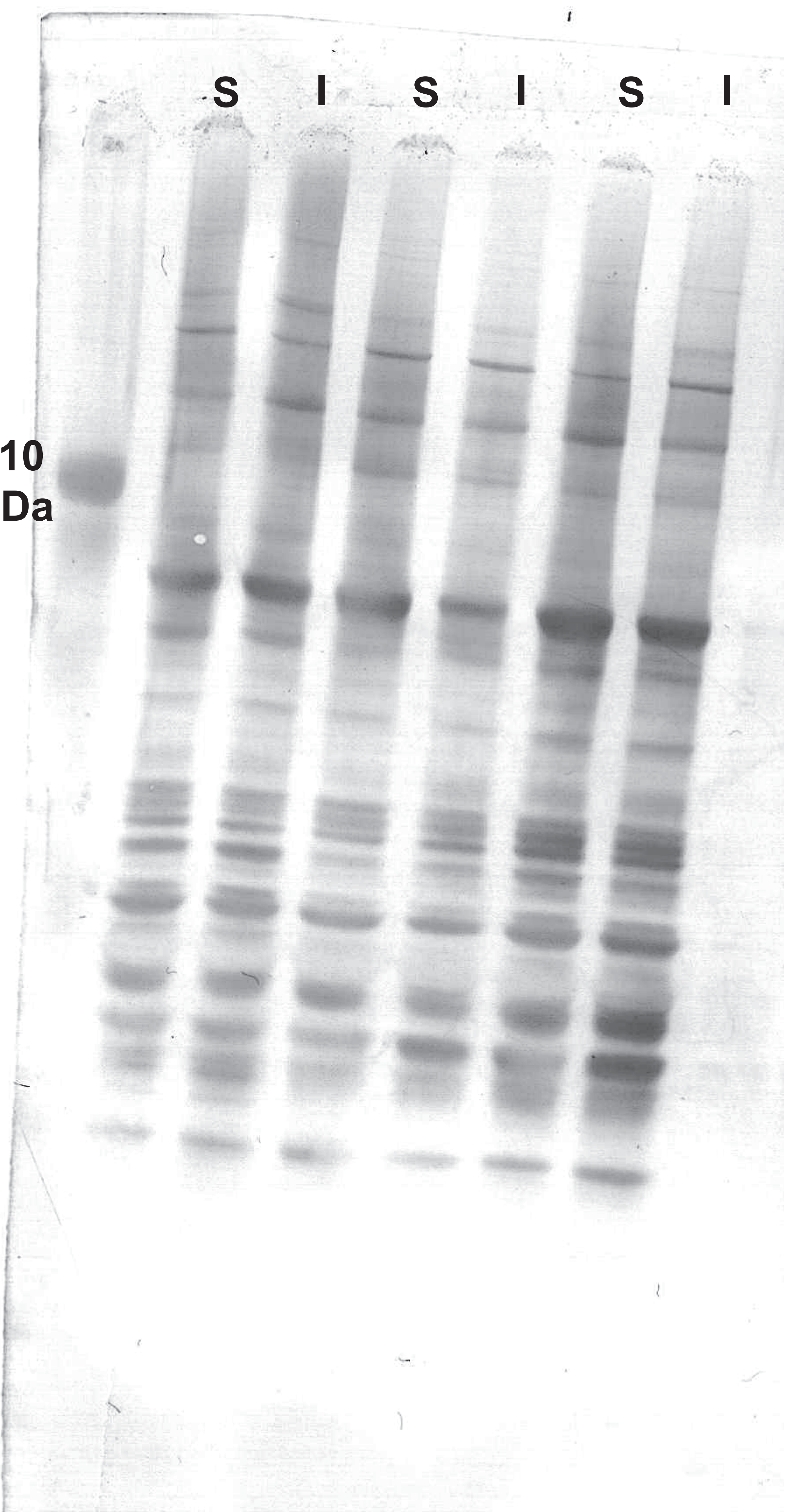

Supplement: Additional file 1: Figure S1. — Coomassie protein staining. Membranes used for western blot analysis of Na+ channels, DHPR (left panel), RyR1and SERCA1 (right panel). Lanes are marked as SHAM (S) or ICU (I); the value 110 kDa refers to the protein marker size in the ladder lane (PDF 5931 kb) [file 13054_2016_1417_MOESM1_ESM.pdf]
