# Supplementary material for: Impaired Ca2+ release contributes to muscle weakness in a rat model of critical illness myopathy
Source: Crit Care. 2016 Aug 10;20:254. doi: 10.1186/s13054-016-1417-z (PMC5050561; doi:10.1186/s13054-016-1417-z)
Supplement: Additional file 2: Figure S2. — Typical examples of the negative controls obtained for Na+ channels, DHPR, RyR and SERCA1 immunohistochemical staining (PDF 871 kb) [file 13054_2016_1417_MOESM2_ESM.pdf]

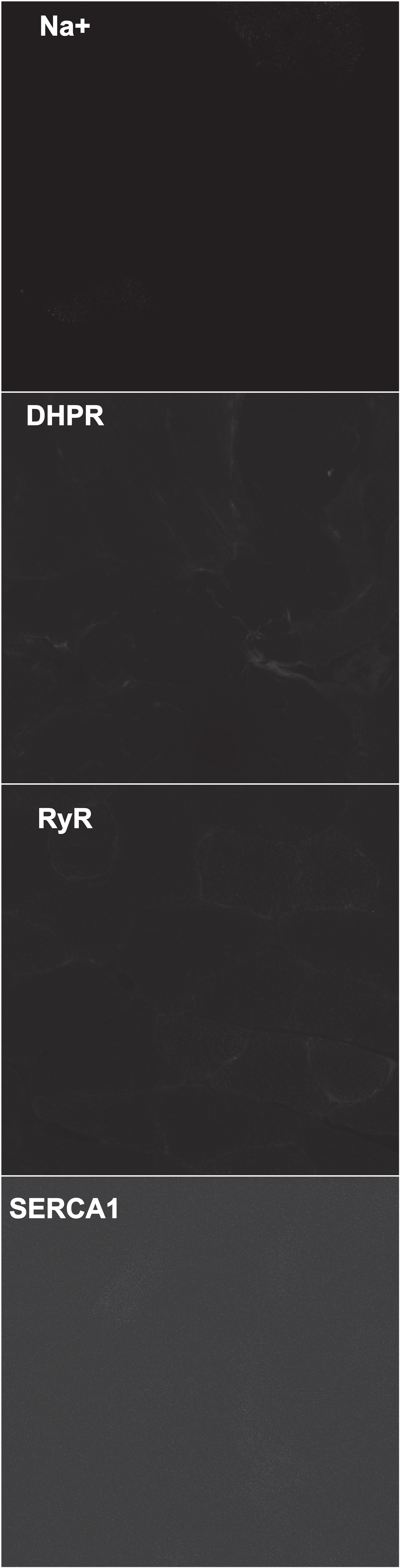
Na+
DHPR
RyR
SERCA1
